# New Screening Test Improves Detection of Prostate Cancer Using Circulating Tumor Cells and Prostate-Specific Markers

**DOI:** 10.3389/fonc.2020.00582

**Published:** 2020-04-23

**Authors:** Karin Ried, Tasnuva Tamanna, Sonja Matthews, Peter Eng, Avni Sali

**Affiliations:** ^1^National Institute of Integrative Medicine (NIIM), Melbourne, VIC, Australia; ^2^Department of Health, Torrens University, Melbourne, VIC, Australia; ^3^Discipline of General Practice, The University of Adelaide, Adelaide, SA, Australia

**Keywords:** prostate cancer, circulating tumor cells (CTC), prostate specific antigen (PSA), early detection, cancer screening

## Abstract

The current screening-test for prostate cancer, affecting 10% of men worldwide, has a high false negative rate and a low true positive rate. A more reliable screening test is needed. Circulating-Tumor-Cells (CTC) provide a biomarker for early carcinogenesis, cancer progression and treatment effectiveness. The cytology-based ISET®-CTC Test is a clinically validated blood test with high sensitivity and specificity. This study aimed to evaluate the ISET®-CTC test combined with prostate-specific-marker staining as a screening test for the detection of prostate cancer. We selected a group of 47 men from our ongoing CTC screening study involving 2,000 patient-tests from Sep-2014 to July-2019, who also underwent standard diagnostic cancer testing before or after CTC testing. While 20 of the 47 men were diagnosed with prostate cancer before the ISET®-CTC test, 27 men underwent screening. We studied the CTC identified in 45 CTC-positive men by Immuno-Cyto-Chemistry (ICC) assays with the prostate-specific-marker PSA. CTC were ICC-PSA-marker positive in all men diagnosed with primary prostate cancer (*n* = 20). Secondary cancers were detected in 63% (*n* = 7/11) of men with mixed CTC-population (ICC-PSA-positive/ICC-PSA-negative). Of the 27 men screened, 25 had CTC, and 84% of those (*n* = 20) were positive for the prostate-specific-PSA-marker. Follow-up testing suggested suspected prostate cancer in 20/20 men by a positive PSMA-PET scan, and biopsies performed in 45% (*n* = 9/20) men confirmed the diagnosis of early prostate cancer. Kidney cancer or B-cell lymphoma were detected in two men with ICC-PSA-marker negative CTC. Our study suggests that the combination of ISET®-CTC and ICC-PSA-marker-testing has an estimated positive-predictive-value (PPV) of 99% and a negative-predictive-value (NPV) of 97%, providing a more reliable screening test for prostate cancer than the standard PSA-blood-test (PPV = 25%; NPV = 15.5%). Our findings warrant further studies to evaluate the new test's potential for prostate cancer screening on a population level.

## Introduction

Prostate cancer is the most common in men, and the second leading cause of cancer deaths (25%) in Australia (1). One in seven men (14%) will be diagnosed with prostate cancer in their lifetime worldwide (1, 2). In 2018, 22.5% men lived with prostate cancer, and >3.7% deaths were attributable to prostate cancer in Australia (1, 2).

The prostate-specific-antigen (PSA) blood test was originally approved by the US-Food-and-Drug-Administration in 1986 to monitor the progression of prostate cancer in men who had already been diagnosed with the disease (2) As a screening-test for men from the age-of-50-years, the PSA-blood-test in conjunction with the digital-rectal-exam has also received support from Medical-Benefit-Schemes and was routinely recommended from 1994 to 2008 (2).

Rising PSA levels are not always associated with prostate cancer, as several non-cancerous conditions, i.e., prostatitis and benign-prostatic-hyperplasia may increase PSA levels in the blood, and can result in false positives (3). In fact, only 25% of men with elevated PSA levels were found to have prostate cancer, leading to unnecessary and potentially harmful follow-up tests such as biopsies in 75% of the men (4). Of these men, 12% experienced bothersome symptoms, including pain and bleeding, or serious infections with 6.9% requiring hospitalization (2, 5).

In addition, a large study with 18,882 men found that routine screening for prostate cancer by the PSA-blood-test and digital-rectal-examination failed to detect prostate cancer in a large proportion (85.5% false-negatives) of screened men with PSA levels below <4.0 ng/ml (3). Three-quarters (78%) of these men had high grade (Gleason ≥ 7) tumors of the prostate (3).

The low-sensitivity (25% true-positives) and low-specificity (14.5% true-negatives) of the PSA-blood-test led to the recommendation against this test as a routine screening-test (6), highlighting the need for a more accurate screening-test for prostate cancer.

To date, modifications of the PSA-blood-test and its interpretation to improve the sensitivity and specificity to better predict cancer have failed (2). Suggested alternative screening methods included free-to-total-PSA-ratio, age-specific-reference-ranges, isoforms-of-PSA, and precursors-of-PSA (2). The PSA-blood-test may however be useful in patients with diagnosed prostate cancer with or without prostatectomy to monitor relapse, indicated by a rising PSA-blood-level over time (7).

Furthermore, current conventional treatment options for prostate cancer, such as surgery and radiation have a high burden of serious complications, including urinary incontinence, bowel and erectile dysfunction, and infection. Specifically, 80% of men diagnosed with prostate cancer undergo a prostatectomy and/or receive radiation therapy, with 60% reporting serious complications (8). Late detection of prostate cancer avoided only one death in 1,000 men (0.1%) (8). Consequently, physicians often recommend a watch-and-wait-approach.

In contrast, our group and others have found that non-invasive blood tests for Circulating-Tumor-Cells (CTC) can help with the early detection of cancer (9, 10), as CTC have been associated with early carcinogenesis and cancer risk (10–12). A meta-analysis on the prognostic role of CTC and prostate cancer specifically involving 33 clinical trials and 4,170 patients suggested a significant association between CTC count and overall survival, biochemical relapse-free survival/disease free survival (HR (95% CI): 2.43 [2.07, 2.86]; 2.15 [1.69, 2.73]; *p* < 0.001) (13).

Several technologies have been developed to identify CTC, including the Isolation-by-SizE-of-Tumor-Cells (ISET®)-CTC-test (Rarecells-Diagnostics, France) (14). The ISET®-CTC-test is a cytology-based clinically validated blood test with high sensitivity and specificity (15–17).

Cytology is an established technique in the field of cancer diagnostics and has been for over a century, with the first microscopic discovery of cancer cells in 1838, and the first monograph of clinical cytology published in 1960s (18, 19). It is now an invaluable specialty in pathology laboratories worldwide and is routinely used as a first line of investigation in cancer diagnostics.

The cytology-based ISET®-CTC-test can distinguish cancer cells from benign cells, using the same cytological criteria as used in routine cancer diagnostics, including anisonucleosis, enlarged nuclei, high nuclear-cytoplasmic-ratio, and irregular nuclear borders (15, 16). Comprehensive analyses of CTC isolated with the ISET® technique, including spiking experiments and genetic testing, verified that all CTC in a blood sample are captured, and had the features of malignant cancer cells (17).

The cytology-based ISET®-CTC-test is independent of the presence of any tumor-surface-markers on cancer cells, such as the Epithelial-Cell-Adhesion-Molecule (EpCAM) markers, used by most other CTC technologies (20, 21). In fact, the ISET®-CTC-test can detect CTC of all cancer types, including epithelial and non-epithelial tumors (22), can distinguish between single-CTC and CTC-clusters (23, 24), with CTC-clusters having greater metastasing potential associated with shorter overall survival (25).

Marker-based CTC-tests have an implicit high risk of false negative results, as cancer cells undergo frequent changes in protein expression and have the potential to lose surface markers, often in later stages of cancer (16). Marker-based CTC-tests can also lead to false-positive-results, as many tumor-markers can be expressed on non-pathological cells, including EPCAM and other markers (26–29).

While it had been recognized that CTC were better prognostic biomarkers than the PSA blood levels for prostate cancer, the CTC detection rate of 57–62% using the marker-based CTC test CellSearch® was considered non-optimal (30, 31). In contrast, the cytology-based ISET®-CTC-test has proven to be of high sensitivity (17) and high specificity (10, 23, 32, 33), and is therefore superior to marker-based CTC-tests in detecting true CTC (16). The ISET®-CTC-test can find one CTC/10 ml of blood, corresponding to 500 CTC/5L of blood in an adult, which is extremely sensitive (17).

While biopsy is the gold-standard technique in the diagnosis of prostate cancer, advances in imaging technologies have been developed to improve the detection of cancer. Specifically, the Gallium-68-prostate-specific-membrane-antigen-positron-emission-tomography (Ga^68^-PSMA-PET) scan is a highly sensitive imaging-test, which can detect tumors as small as 2.4 mm, and has shown promise in enhancing the detection and localization of prostate cancer (34, 35).

In a study of men with persistently elevated prostate PSA levels but negative digital rectal examination and negative biopsy, Ga^68^ PSMA-PET guided biopsy was found to be useful in identifying clinically significant primary prostate cancer (Gleason ≥ 7), with 100% overall sensitivity, and 76–88% specificity (36).

The superior diagnostic accuracy of the PSMA-PET scan compared to MRI and PET imaging alone in the detection of primary prostate cancer, its usefulness in screening, as well as guiding biopsies have been described in several independent studies (37–39).

Continuous screening and monitoring are also relevant for patients previously diagnosed with cancer, as recurrences or relapses of their cancer may be more challenging to treat, and timely detection may assist with recovery. In this context, the accuracy of a screening/diagnostic test for recurrent cancer is as important as for the early detection of primary cancer.

A study of 42 patients concluded that the PSMA-PET scan was helpful in detecting prostate cancer in 83% of patients with suspected recurrent cancer, while false positive lesions were not detected (40). Furthermore, a large Australian study of 431 patients with prostate cancer found the PSMA-PET scan to be a valuable diagnostic tool in the management of patients with prostate cancer, as the uptake of ^68^GA-labeled PSMA marker allowed the detection of unsuspected disease in the prostate bed, local lymph nodes, and distant metastases, which had not been detected by other imaging techniques. The study found that the PSMA-PET scan reduced the need for bone scintigraphy, CT scans, MRI, and ^18^F-FDG PET scans (41).

The high sensitivity (near 100%) and high specificity (about 80%) of the PSMA-PET scan demonstrated in several studies, makes it the excellent non-invasive tool for the detection of prostate cancer.

In our previous study with 2,000 patients, CTC were detected in 50% and early cancer in 25%, including prostate cancer (9). The prostate cancer diagnosis was facilitated by the PSMA-PET scan, followed by biopsy in a proportion of men (9).

PSA-markers are found in cells of prostate origin (99% specificity), and are less expressed in kidney, parotid gland and pancreas (42). Prostein (also known as P501S) markers are only in prostatic epithelial cells (100% specificity; 100% sensitivity) (43). A small proportion (3%) of men have no PSA-markers on prostate cells, providing a 97% sensitivity (3, 43, 44).

In this study we combined the ISET®-CTC screening blood test and immuno-cyto-chemistry (ICC) with prostate-specific markers in men with prostate cancer and in men screened for prostate cancer. Screened men with a positive CTC count and prostate-marker positive CTC were followed up with PSMA-PET scan and/or biopsy for prostate-specific diagnostic testing.

## Materials and Methods

### Study Design and Participants

For this observational study, doctors and health practitioners mainly from two medical clinics in Melbourne, Australia, the National-Institute-of-Integrative-Medicine Clinic, and the Eng-Medical-Center, referred patients to the ISET®-CTC-test between Sep-2014 and Jul-2019. Follow-up of participants was coordinated by the patients' doctors as per usual clinical practice. Routine and follow-up testing included PSA-blood-tests, PSMA-PET scans, MRI, biopsies, and any other diagnostic tests, according to the patient's doctor advice.

The study was approved by the NHMRC-endorsed NIIM Human-Research-Ethics-Committee. Participating patients provided written informed consent. The study has been registered on the Australian-New-Zealand-Clinical-Trial-Registry, ANZCTR 12614001143617.

### Inclusion Criteria and Selection of Patient Samples for ICC Prostate-Specific Analysis

For this study, we selected two groups of men from our ongoing CTC study involving 2,000 patients-tests (9), who had also standard cancer diagnostic test results available, either before (group PC) or after (group ED) CTC testing. CTC blood test samples for ICC analysis from the first group of men diagnosed with prostate cancer *before* CTC analysis were considered as positive control. The second group of men underwent follow-up diagnostic testing *after* the CTC screening blood test. As negative control for the prostate-specific markers, we undertook ICC-analysis on CTC of two female cancer patients.

### Circulating Tumor Cell (CTC) Detection by ISET®-CTC Test

In this study, we used the ISET®-CTC methodology combining blood filtration and microscopic analysis using standard cyto-pathological criteria, validated in the over 70 peer-reviewed publications over 20 years (16, 32). We followed standardized validated protocols described previously (15–17).

Briefly, the ISET® method is a blood filtration-based approach, which enriches rare cells on a polycarbonate membrane with 8 μm pores. Ten milliliter of peripheral blood was collected in buffered EDTA, maintained at room temperature (RT) and processed within 2 h of collection. Blood was then diluted 1:10 with Rarecells® Buffer reagent set (Rarecells Diagnostics, France) containing saponin, salt and bovine serum albumin, agitated for 10 min at RT, and filtered with the ISET® filtration blocks and device (15).

The ISET®-CTC-test can detect CTC of all cancer types, including epithelial and non-epithelial tumors. Human cancer cells are larger than the 8 microns filter pore size (22). In fact, CTC range from 11.7 to 23.8 microns for solid tumor cells, 7.2–10 microns for small-cell type cancers (e.g., small cell lung carcinoma) and 8.9–15.3 microns for blood type cancers.

The dried filter membrane was stained with May-Gruenwald-Giemsa for cytological analysis. A cytologist, with Australian and International cytology certification [CT(ASC), CT(IAC)] and more than 25 years' experience conducted the analysis using a Leica DMLB microscope with 63 × 10 magnification and standard cytological criteria to identify malignant cells.

Circulating malignant cells were defined by the presence of 4 of the following criteria: (a) anisonucleosis (ratio > 0.5), (b) nuclei larger than 1–3 calibrated pore sizes (8 μm) of the membrane (i.e., >8–24 microns), (c) irregular nuclear borders, (d) high nuclear-cytoplasmic ratio, and/or (e) presence of three-dimensional sheets. Cells displaying 1–3 criteria were defined as atypical cells with uncertain malignant potential. Circulating benign cells were characterized by the absence of these criteria (16).

Images of CTC and atypical cells were taken with a digital Leica EC3 camera, reviewed independently by a second cytologist / researcher and any discrepancies were discussed.

### Immuno-Cyto-Chemistry (ICC) Staining With Prostate-Specific Antibodies

ICC staining was conducted with the Dako EnVision Flex Mini Kit, high pH, and antibodies (a) PSA (Dako monoclonal mouse anti-human prostate-specific antigen Clone ER-PR8, concentrate), and (b) Prostein/P501S [Dako Flex Monoclonal mouse anti-human Prostein Clone 10E3, Ready-to-Use (RTU)].

For antigen retrieval, ISET® filters were placed into a 50 ml tube and heated in a water bath at 99°C for 40 min in 50 mL of 2% v/v target retrieval solution (high pH (50x): 3 × 30 mL, 50x concentrate Tris/EDTA buffer, pH9; Dako Agilent). Next, the ISET® filters were washed 15x in 0.2 M PBS and incubated with permeabilization buffer (0.1% Triton) for 2 min. After washing the ISET® filter as before, the ISET® filter was incubated for 30 min with peroxidase-blocking reagent (80 μL of H_2_O_2_, 15 mmol/L Na_3_N and detergent), and washed with distilled water 15 times.

Each ISET®-filter spot was incubated overnight at 4°C in 600 μL solution of the primary antibody (a) 1:200 dilution of concentrated PSA antibody or (b) 1:4 dilution of Prostein antibody RTU in a buffer of 1xPBS / 5%BSA in a 1.5 ml Eppendorf tube.

After incubation with the primary antibody, ISET®-filter spots were washed in PBS as before, placed on a clean slide, for incubation with 80 μL of the secondary antibody (Dako Envision Flex Kit goat secondary antibody) for 45 min at RT.

The ISET®-filter spots were washed again, before incubation with 80 μL of chromogen (Dako Liquid DAB + Substrate Chromogen (3,3 diaminobenzidine tetrahydrochloride) on a glass slide in the dark for 10 min. The excess chromogen was removed by washing in 0.2 M PBS. The filter spots were allowed to air-dry, then counterstained with hematoxylin for 2 min. After washing in PBS as before, each air-dried immuno-stained spot was mounted on a clean glass slide for microscopic analysis.

## Results

**Figure 1:**
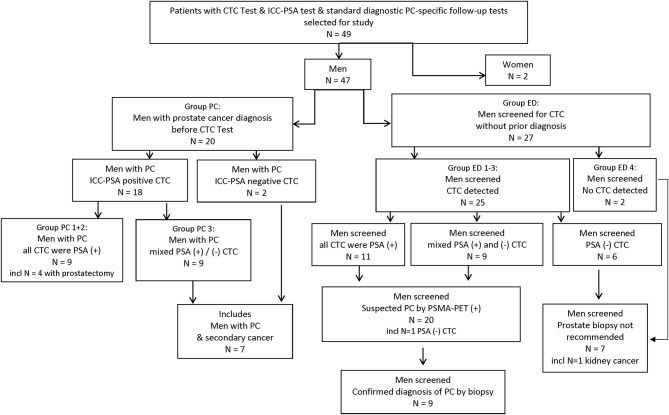
Study flow chart. CTC, circulating tumor cell; ED, early detection; ICC, immuno-cyto-chemistry; incl, including; PC, prostate cancer; PSA, prostate specific Antigen; (+)/(–), positive/negative test result.

**Figure 2:**
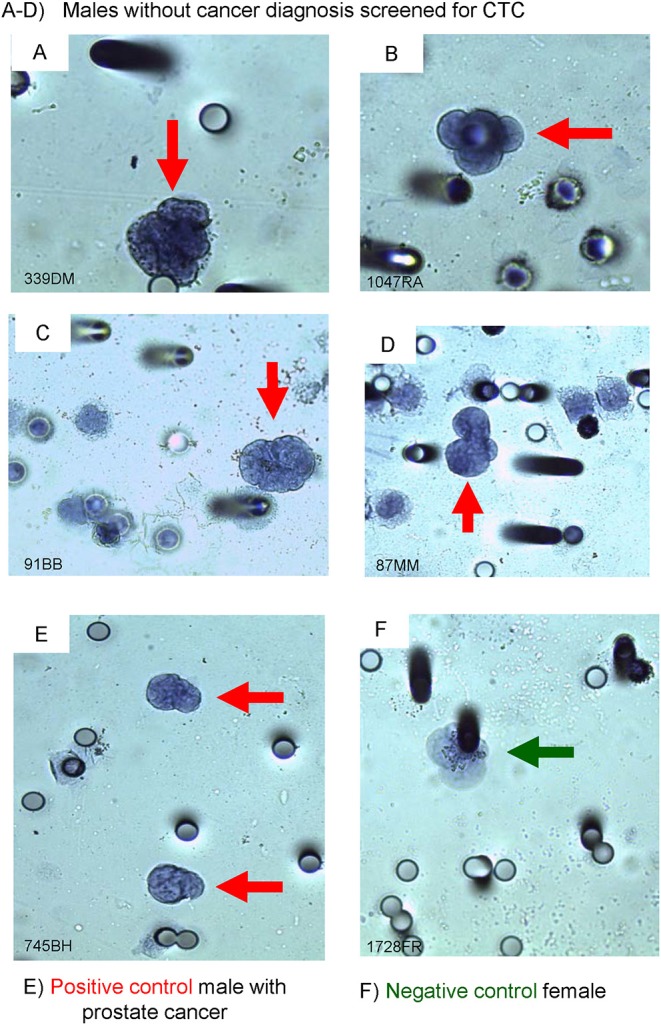
Immuno-cyto-chemistry (ICC) on ISET®-CTC with PSA-antibody. **(A–D)** screened males with PSA (+) stained CTC, **(E)** male with PROSTATE CANCER (positive control), and **(F)** a female breast cancer patient (negative control). The black outline of the Circulating Tumor Cell (CTC) (red arrows) depicts a positive ICC marker stain, no black outline around the CTC depicts a negative ICC marker stain (green arrow).

A total of 49 patient samples were selected for this study. These included samples from 47 men and 2 women (Figures 1, 2).

### Prostate Cancer Patients (Group PC)

In a group of 20 men with diagnosed prostate cancer and positive CTC count, we tested CTC cells for ICC-PSA-markers. Of the men aged 50–80 years (mean 65 years), the CTC-count ranged between 1 and 23 CTC/ml (mean 6.5 CTC/ml) at 1 month to 12 years after prostate cancer diagnosis (Table 1).

**Table 1:** ISET®-CTC and ICC prostate marker test results AFTER diagnosis in patients with prostate cancer (group PC).

**Test ID**	**Group**	**Age range at CTC test**	**PSA** **μg/L (date)**	**CTC number/mL**	**Date CTC test**	**ICC-PSA marker (+) % of CTC**	**Time between diagnosis and CTC test**	**Prostate cancer diagnostic tests (date)**	**Treatment between diagnosis and CTC test**	**Comments**
**Group PC 1: Prostate cancer patients; ICC-PSA marker (+) 100%**										
14GC;	PC with bone mets	65–69 yrs	319 (Aug-14); 618 (Sep-14); 1531 (Dec-14);	180 CTC/mL incl clusters	Dec-14		1 yr	Biopsy (+) (2005) MRI: bony metastasis (Dec-13)	Had 2nd chemo and weekly hyperthermia (8/14–9/15)	
38GC;			561 (Jan-15); 385 (Feb-15);	17.1 CTC/mL	Jan-15					
113GC			552 (Mar-15)	6.7 CTC/mL	Mar-15	100%			Died 10/15	
813CM	PC	70–74 yrs	2.65 (Jul-18)	1.5 CTC/mL	Jan-17	100%	1 mth; 2.5 yrs after CTC	Biopsy (+); MRI (+) (Dec-16) PSMA-PET (+) (Jul-19)	Integrative nutritional therapies incl supplements	Gleason 6, +4 mm pulmonary nodule; MRI-PI-RADS
964BH;	PC	65–69 yrs	5.5 (Aug-16);	2.9 CTC/mL	Mar-17		4 mths	Biopsy (+) (Nov-16)	Sonotherapy daily for 2 years, supplements	PC adenocarcinoma localized
1885BH;			3.1 (May-19); 3.7 (Jun-19); 4.6 (Jul-19); 5.0 (Aug-19);	0.2 CTC/mL	May-19	100%	2.5 yrs		Hyperthermia 12 sessions, HBOT +IVC (Nov-18),	
2002BH			3.3 (Sep-19)	0.8 CTC/ml	Sep-19					
1423FC	PC	80–84 yrs	3.2 (Feb-19)	2.6 CTC/mL	May-18	100%	7yrs	Biopsy (+) (2011)	IVC and hyperthermia 2x 2012; Supplements 2012–2018	Gleason 6
12FP	PC	70–74 yrs	nk	1 CTC/mL	Sep-14	100%	1 yr	Biopsy (+); MRI (+) (2013)	No treatment	Stage 3
**Group PC 2: Prostate cancer patients with prostatectomy; ICC-PSA marker (+) 100%**										
46PB	PC	55–59 yrs	0.121 (Sep-15)	1.7 CTC/mL	Dec-14	100%	10mths	PC: Biopsy (+) CT: Bone mets (Mar-14) PC: PSMA-PET (+) (Aug-17)	Radiotherapy (May-14) Prostatectomy (Jun-14) Hyperthermia /IVC (Jul-14)	ICC marker on CTC: 100% PSA (+) and 0% Prostein (+)
1638AL	PC	60–64 yrs	15.7 (Sep-11); 23.2 (Dec-17); 0.01 ^#p^ (Nov-18)	1.1 CTC/mL	Nov-18	100%	11 mths	Biopsy (+); PMSA-PET (+) (Dec-17)	Prostatectomy (Dec-17) Chemotherapy Jan-Jun-18 (hormone injection)	Stage 4
561GK	PC	60–64 yrs	0.066 ^#p^ (May-15); 6.0 (May-16)	2.0 CTC/mL	Jun-16	100%	1 yr	Biopsy (+) (May-15)	Radical prostatectomy (May-15)	Gleason 3 + 4 = 7, bilateral PC adenocarcinoma
JM;	PC	70–74 yrs	3.9 (2011); 7 (2012); 9.6 (2013); 12.3 (2014); 17.9 (2015);					Biopsy (+) (2010)	Prostatectomy (Nov-2015); Integrative nutritional therapies	ICC marker on CTC: 100% PSA (+) and 50% Prostein (+)
1041JM			21.9 (2016)	23.3 CTC/mL	May-17	100%	7yrs			
**Group PC 3: Prostate cancer patients; ICC-PSA marker (+)** ** <100%**										
238NM; 357NM; 534NM	PC	65-69 yrs	4.3 (Jul-15); 4.2 (Sep-15); 6.0 (May-16)	2.0 CTC/ml 1.5 CTC/ml 6.2 CTC/mL	Jul-15 Nov-15 May-16	28%	1 mth 5 mths 11 mths	**NHL**: bone marrow aspirate (Feb-14); PC: MRI/ biopsy (+) (Apr-15)	Integrative nutritional therapies, CB	PIRADS 4 bilateral adenocarcinoma, 3+3 Gleason **NHL** **=** **Non-Hodgkin's lymphoma**
JP;	PC (2007)	70-74 yrs	4.6 (2007); 3.6 (2008); 4.3 (2009); 5.7 (2010); 6.9 (2011); 5.6 (2012); 4.8 (2013);					PC: MRI (+) (2007); Biopsy (+) (Jul-12)	Sonotherapy, integrative nutritional therapies	MRI with spectroscopy (2007), TRUS biopsy (2012)
11JP;			14.6 (Sep-14);	2.5 CTC/ml	Sep-14		7 yrs	Biopsy (+)		
421JP; 1097JP; 1391JP; 1598JP;			7.8 (2015); 5.5 (2016); 7.3 (2017); 6.0 (2018);	0.2 CTC/ml CTC/ml CTC/ml 0.4 CTC/ml	Feb-16 Jun-17 Mar-18 Oct-18					
1791JP;	+ skin (2019)		7.0 (Oct-19)	4.5 CTC/mL	Feb-19	40%	12 yrs	+Skin ca (Feb-19)		**Keratoacanthoma** (variant SCC; skin cancer)
17IK	PC	75-79 yrs	113 (2014); 103(2015); 106 (2016);	1.4 CTC/mL	Oct-14	50%	3 yrs	MRI (+) (2011+2012); Biopsy (+) (2013)	Sonotherapy, integrative nutritional therapies	Bilateral adenocarcinoma SD: 2011/2012
			153 (2017); 181 (2018)					+Mets lymph nodes (PET-CT scan Jan-17)		**Metastases Lymph Nodes** Jan-17; Died Apr-19
745BH	PC + bone mets	65-69 yrs	2500 (Nov-16)	23.3 CTC/ml	Nov-16	66%	3 yrs	PC: Biopsy (Nov-13); Bone mets: PET (Nov-16)	Radical prostectomy (Jan-14); Hyperthermia + IV × 10 (2016–2017) after CTC test	**Metastases Bone** Nov-16
509GA; 780GA; 1042GA; 1137GA; 1304GA; 1411GA; 1607GA;	PC	65-69 yrs;	1.8 (2016);	2.8 CTC/mL CTC/ml 3.2 CTC/ml 0.8 CTC/ml 1.2 CTC/ml 0.3 CTC/ml 0.5 CTC/ml	May-16 Dec-16 May-17 Jul-17 Dec-17 Apr-18 Oct-18		1 yr;	**Bladder:** MRI (+) (2014); PC: Biopsy (Sep-15); PSMA-PET (–) (Jan-16) after #prostatectomy	#Prostatectomy Jan 16	**Bladder (high grade urothelial carcinoma)**, PC (highest grade adenocarcinoma)
1949GA		70-74 yrs	0.01^#p^ (Jun-19)	2.4 CTC/mL	Jul-19	11%	5 yrs			CTC test after prostatectomy
1430GE	Thyroid; PC	55-59 yrs	1.03 (2016)	1.5 CTC/mL	May-18	<15%	2 yrs;	**Thyroid:** Biopsy (+) (2016) **PC:** PSMA-PET (+) (Mar-16); Biopsy (+)	Thyroidectomy (Jun-16); Integrative nutritional therapies for PC	CTC 2 yrs after diagn; CTC likely not prostate, but may be related to **thyroid** cancer
1858JW	Primary tongue; 2^nd^ PC	80-84 yrs	0.84 (May-19)	4.9 CTC/mL	Apr-19	0%	3 yrs	**Tongue:** MRI (+) (2016); **PC:** PSMA-PET (+) (10/16); Biopsy (+)	Neck surgery/tongue dissection (2016); Integrative nutritional therapies	CTC test 3 yrs after diagn likely not prostate, but may be related to **tongue** cancer
1834PK	PC + bowel, bladder mets	50-54 yrs	0.2 (2019)	6.4 CTC/mL	Apr-19	0%	12mths	Biopsy (+) (Apr-18)	Prostatectomy + hemi-colectomy (2018); Hyperthermia + IVC AFTER CTC test	CTC likely non-prostate origin, but may be **bowel, bladder**
61DT;	PC	70-74 yrs;	0.73 (2013)	3.1 CTC/mL	Dec-14;	0%	1.5 yrs;	Biopsy (+) (Nov-12)	Prostatectomy (2013);	TURIP
1953DT		75-79 yrs	1.13 (2016)	0.9 CTC/ml	Jul-19	20%	6 yrs	PSMA-PET (–) (Aug-16)	Integrative and nutritional therapies	
490DM	PC	75-79 yrs	2.02 (Sep-16)	5.4 CTC/mL	Apr-16	10%	3.5 yrs	Biopsy (+) (Nov-12); PSMA-PET (+) (Dec-16)	Stereotactic radiotherapy, sonotherapy, integrative nutritional therapies	ICC markers on CTC: 10% PSA (+) and 6% Prostein (+)
1894RB	PC	70-74 yrs	16.8 (May-19)	4.8 CTC/mL	May-19	20%	6 mths	Biopsy (+) (Nov-17)	Hyperthermia, IVC + supplements	2 cell populations, 20% PC, 80% may be of different origin; ICC markers on CTC: 20% PSA (+) and 50% Prostein (+)

*CTC, circulating tumor cells; ca, cancer; HBOT, hyperbaric oxygen; IVC, intravenous vitamin C; ICC, immune-cyto-chemistry; ISET®, Isolation by SizE of Tumor Cells; PC, prostate cancer; PSA, prostate specific antigen; PSMA, prostate specific membrane antigen; PET, positron emission tomography; mets, metastasis; mL, milliliter; mths, months; na, not applicable; nk, not known; #p, after prostatectomy; PSA (+), PSA positive CTC; PSA (–), PSA negative CTC; yrs, years; (+)/(–), positive/negative test result. Bold: cancer diagnosis other than prostate cancer*.

PSA-blood-test results were ≤ 5 ng/ml in two-thirds of men (65%, 13/20) while 35% (7/20) showed elevated PSA-levels at time of diagnosis or CTC testing (Table 1).

The ICC-PSA-marker test was positive in all men diagnosed with prostate cancer (90%, *n* = 18), except in two cases with secondary cancers (oral or colon/bladder) and/or prostatectomy.

Forty-five percent of the men with prostate cancer (9/20) demonstrated 100% positivity for PSA-markers, while 55% had a heterogeneous population of cancer cells (ICC-PSA-positive-CTC and ICC-PSA-negative-CTC) (Figure 1, Table 1).

In two-thirds of the cases with mixed CTC populations or ICC-PSA-negative-CTC (66%, 8/12), secondary cancer or metastases were diagnosed within 1-to-12 years after prostate cancer diagnosis, and included bladder, bowel, skin, Non-Hodgkin's-Lymphoma, thyroid, tongue cancer, and lymph node involvement (Table 1).

The ICC-PSA-negative-CTC may represent cells from the secondary cancers in these patients. However, other explanations such as lower expression of PSA antigen on CTC or loss by mutation cannot be ruled out.

### Early Detection of Cancer in Screened Patients (Group ED)

The second group of men we selected for this study had also standard cancer diagnostic test results available *after* CTC screening (group ED). Out of the selected 27 men without prior cancer diagnosis, 25 men were positive for CTC. The majority of these were also positive for ICC-PSA-markers (80%, 20/25). Three-quarters of men screened with CTC (80%, 20/25) had prostate-specific diagnostic follow-up tests, and 20% (5/25) had other follow-up diagnostic testing within 1 month to 3.5 years of the CTC-test (Figure 1, Table 2, group ED1+2).

**Table 2:** ISET®-CTC and ICC-prostate marker test results of men not previously diagnosed with cancer—Group “early detection” (ED).

**Test ID**	**Group**	**Age range at CTC test**	**PSA** **μg/L (date)**	**CTC/mL**	**CTC test (date)**	**ICC PSA** **marker (+) in % of CTC**	**Time between CTC test and diagnosis**	**PC diagnostic test by biopsy (date) AFTER CTC test**	**Other tests (date) after CTC test**	**Treatment**	**Comments**
**Group ED 1: Early detection of PC: ICC PSA marker (+) in 100% of CTC tested and prostate biopsy (+)**											
133JW;	Screen	60–64 yrs	nk	2.2 CTC/mL	(Apr-15)	100%	3.5 yrs			No treatment	ICC marker on CTC: 100% PSA (+) and 100% Prostein (+)
1916JW	PC		8,803 (Sep-18)					Biopsy (+) (Sep-18)	PET-CT: PC with bony metastasis (Sep-18)		
55NZ;	Screen	55–59 yrs	1.44 (Jun-15)	2.6 CTC/mL	(Dec-14)	100%	6 mths		PSMA-PET (+) (Jun-15)		
535NZ;	PC			4.6 CTC/mL	(Jun-16)					Hyperthermia x20 (2015/16)	
907NZ; 1232NZ; 1330NZ; 1506NZ;			0.01 ^after#p^ (Oct-17)	2.4 CTC/mL 6.5 CTC/ml 0.6 CTC/mL 0.1 CTC/mL	(Feb-17) (Oct-17) (Jan-18) (Aug-18)			Biopsy (+) (Feb-17)		#Prostatectomy (2017); metformin, NT	
517SM	Screen; PC	45–49 yrs	3.9	1.3 CTC/mL	(May-16)	100%	1 mth	Biopsy (+) (Apr-16)	PSMA-PET (+) (Apr-16)		PC adenocarcinoma involving 5 sites, Gleason 7
86BD	Screen; PC	60–64 yrs	0.01 after #p (Mar-16)	4.1 CTC/mL	(Feb-15)	100%	6 mths	Biopsy (+) (Aug-15)		#Prostatectomy (Aug-15)	
123RM	Screen; PC	65–69 yrs	6.4	5.5 CTC/mL	(Mar-15)	100%	5 mths	Biopsy (+) (Aug-15)	PSMA-PET (+) (Aug-15)	Radical #prostatectomy (Nov-15)	
87MH	Screen; PC	50–54 yrs	1.77	4.0 CTC/mL	(Feb-15)	83%	6 mths	Biopsy (+) (Aug-19)	PSMA-PET (+) (Aug-15)	Radical #prostatectomy (Aug-19)	MRI normal (2015)
92AG 1595AG; 1919AG;	Screen	70–74 yrs	1.97	3.1 CTC/mL 73.3 CTC/mL 13.7 CTC/mL incl cluster	(Feb-15) (Oct-18) (Jun-19)	100%	8 mths	Biopsy (+) (Oct-18)	PSMA-PET (+) (Oct-15)	IVC 20x (2015)	ICC marker on CTC: 100% PSA (+) and 100% Prostein (+)
1664AC	Screen	55–59 yrs	5 (Nov-18); 6.1 (Nov-19)	11.1 CTC/mL	(Nov-18)	70%	2 mths	Biopsy (+) (Jan-19)	MRI (+) (Jan-19)	Discussed prostatectomy	Gleason 7 (Jan-19);
91BB	Screen	70–74 yrs		4.4 CTC/mL	(Feb-15)	100%	2yrs			IVC x20 (2015)	ICC marker on CTC: 100% PSA (+) and 100% Prostein (+)
454BB				1.2 CTC/mL	(Mar-16)		1 yr				
1193BB				4.0 CTC/mL	(Apr-17)		1 mth		Multiparametric MRI (+) (May-17)		
1490BB			1.7 (Apr-18)	12.6 CTC/mL	(Jul-18)			Biopsy (+) (Apr-18)	PSMA-PET (+) (Aug-18)	IVC+ hyperthermia (20x 2018)	
1690BB				0.2 CTC/ml	(Dec-18)						Dec-18: CTC after treatment
**Group ED 2a: Suspected PC (75% PPV, 100% NPV): ICC PSA marker (+) in 50-100% of CTC tested, PSMA-PET (+), declined biopsy**											
1527PEj	Screen	45–49 yrs	0.96	39.4 CTC/mL	(Aug-18)		1 mth	Declined biopsy	PSMA-PET (+) (Sep-18)		Mold exposure, high mycotoxin
1709PEj	PC			3.0 CTC/mL	(Dec-18)	100%				10x hyperthermia + IVC + NT	CTC count reduced after treatment
1982PEj				0.5 CTC/mL							
137JS; 649JS; 884JS;	Screen	80–84 yrs		1.5 CTC/ml 1.8 CTC/ml 3.3 CTC/mL	(Apr-15) (Aug-16) (Feb-17)		1yr 9mth				
1579JS;			11.1 (Apr-18)	7.2 CTC/mL	(Oct-18)		1 mth	Declined biopsy	PSMA-PET (+) (Nov-18)	Hyperthermia + NT 6x	
1899JS				0.1 CTC/mL	(May-19)	100%					Lower CTC count after treatment
81LD	Screen	75–79 yrs	2.19	4.9 CTC/mL	(Jan-15)	100%	10 mths	Declined biopsy	PSMA-PET (+) (Oct-15)	NT after CTC test	Mild uptake in both lobes
1930SP	Screen	30–34 yrs	0.5	2.2 CTC/mL	(Jun-19)	100%	1 mth	Declined biopsy	PSMA-PET (+) (Jul-19)		
1047RA	Screen	75–79 yrs	1.37	4.9 CTC/ml	(May-17)	90%	1 yr	Declined biopsy	PSMA-PET (+) (May-18)		MRI (–) 6/2018 Prostein (+) 100%
506GP	Screen	45–49 yrs	0.73	65.4 CTC/mL	(May-16)	83%	1 mth	Declined biopsy	PSMA-PET (+) (Jun-16)		PSMA-PET: Moderate uptake
263EN	Screen	65–69 yrs	1.25 (12/15)	0.6 CTC/mL	(Aug-15)		7 mths	Declined Biopsy	PSMA-PET (+) (Mar-16)		Possible low-grade prostate cancer in left posterior peripheral zone, more concerning uptake in right hepatic lobe
480EN				5.4 CTC/mL	(Apr-16)	84%	−1 mth				
523DC	Screen	65–69 yrs	3.9 (May-16)	10.7 CTC/mL	(May-16)	50%	1 mth	Declined biopsy	PSMA-PET (+) (Jun-16)		PSMA-PET: low to moderate uptake
1962KP	Screen	75–79 yrs	21.7 (Jul-19)	6.2 CTC/mL	(Jul-19)	50%	1 mth	Declined biopsy	PSMA-PET (+) (Aug-19)		Low grade diffuse uptake within enlarged left posterolateral prostate gland, suspicious for non-PSMA avid PC (prevalence 10%)
**Group ED 2b: Suspected PC (75% PPV, 100% NPV): ICC PSA marker (+) in** ** <50% of CTC tested, PSMA-PET (+) and declined biopsy**											
304AB	Screen	65–69 yrs	0.33 (Sep-15)	1.1 CTC/mL	(Sep-15)	14%	1 mth	Declined biopsy	PSMA-PET (+) (Oct-15)		
1940JXX	Screen	60–64 yrs	nk	2.4 CTC/mL	(Jul-19)	0%	1 mth	Declined biopsy	PSMA-PET (+) (Jul-19)		PSA specificity in Asian population 10% compared to 3% in Caucasian
**Group ED 3: ICC PSA marker (+) in 0-10% of CTC tested, prostate biopsy not recommended, incl. non-prostate cancer detected**											
553PF	Screen; kidney	54–59 yrs		7.2 CTC/mL	(Jun-16)	0%	1 mth	Biopsy not recommended	MRI (+): **Kidney** ca (Jul-16)	nephrectomy Jul-2016	ICC prostate marker on CTC: 0% PSA (+) and 0% Prostein (+)
1939GR	Screen	45–49 yrs	0.55 (Jul-19)	15.9 CTC/mL	(Jun-19)	0%	11 mths	Biopsy not recommended	MRI neck (+); Neck biopsy: **B-cell lymphoma:** (Jul-18)		Haemoptysis (coughing up blood), mediastinal mass 2.1 × 1.3 × 3.0 cm; has enlarged adenoids and palatine tonsils
1869SJ	Screen	65–69 yrs	1.9 (May-19)	12.7 CTC/mL	(May-19)	0%	1 mth	Biopsy not recommended	Full body PET-CT scan (–) (Jun-19)		
1966HM	Screen	75–79 yrs	2.09 (Nov-18)	4.3 CTC/mL	(Jul-19)	0%	−2 yrs	Biopsy not recommended	Previous PSMA-PET (–) (Aug-17)		
1519AS	Screen	58 yrs	2.51 (Aug-18)	4.8 CTC/mL	(Aug-18)	0%	1mth	Biopsy not recommended	CT Chest, abdomen, pelvis (–) (Sep-18)		Small hepatic and renal lesions, likely cysts; no lymphadenopathy in the chest, abdomen, pelvis
**Group ED 4: No CTC, but atypical cells and inflammatory cells, follow-up prostatitis**											
312MB	Screen	65–69 yrs	3.7 (Sep-15)	0.5 atypical inflammatory cells/mL	(Sep-15)	na	2.5 mths	Biopsy not recommended	PSMA-PET (–) (Nov-15)	Prostatitis treatment	PSMA-PET: no significant accumulation, no evidence of nodal, or distant metastases; marked prostatomegaly, but no tumor
408PS	Screen	75–79 yrs	3.0 (Feb-16)	0.7 atypical inflammatory cells	(Feb-16)	na	3 mths	Biopsy not recommended	PSMA-PET (–) (May-16)	Prostatitis treatment	
**Group: Negative control—female cancer patients**											
1728FR	Breast ca	70–74 yrs	na	2.3 CTC/mL	(Jan-19)	0%	−6 yrs	N/A	Breast (Jun-13)	na	Negative control
13AB	Ovarian ca	45–49 yrs	na	3.6 CTC/mL	(Sep-14)	0%	<1 mth		Ovarian (Sep-14)	na	Negative control

*CTC, circulating tumor cells; ca, cancer; ICC, immuno-cyto-chemistry; ISET®, Isolation by SizE of Tumor Cells; IVC, intravenous high dose vitamin C; NPV, negative predicted value; NT, nutritional treatment incl supplements; PC (clin diagn), prostate cancer (clinical diagnosis); PPV, positive predictive value; PSA, prostate specific antigen; PSMA, prostate specific membrane antigen; PET, positron emission tomography; mL, milliliter; mths, months; na, not applicable; nk, not known; #p, after prostatectomy; PSA (+), PSA positive CTC; PSA (–), PSA negative CTC; w, weak; yrs, years; (+)/(–), positive/negative test result. Bold: cancer diagnosis other than prostate cancer*.

The average CTC count in the tested samples of men aged 30–83 years (mean = 58 years) was 1–13 CTC/ml (mean = 3.1 CTC/ml), and two patient samples had higher counts of 39 and 65 CTC/ml.

PSA-blood-test results at the time of the CTC-tests were ≤ 5 ng/ml for all but three men (88%, 22/25) (Table 2).

Follow-up prostate-specific diagnostic tests including biopsy were recommended for all men with ICC-PSA-positive-CTC (80%, *n* = 20/25). This group included a man of Asian background with ICC-PSA-negative-CTC, as the specificity of the PSA-marker is lower (90%) in an Asian population compared to 97% in Caucasians (45).

Positive PSMA-PET scan results suggested suspected prostate cancer in all men with ICC-PSA-positive-CTC (*n* = 20). Forty-five percent of these men (*n* = 9/20) agreed to biopsies, which confirmed the diagnosis of early prostate cancer in each one (Figure 1, Table 2).

Prostate-specific biopsy was not recommended in five Caucasian men with ICC-PSA-negative CTC, instead non-prostate specific follow-up tests detected kidney cancer in one patient, and B-cell lymphoma in another, while no other tumors were detected in 3/6 men with ICC-PSA-negative CTC (Table 2, group ED3).

In summary, out of the 27 men in the early detection group (group ED), 25 men had CTC, 2 men had no CTC. Twenty out of the 25 men with CTC had ICC-PSA-positive markers, and all of these 20 (100%) were diagnosed with prostate cancer.

In these subgroups of men the detection of CTC by the cytology-based ISET methodology matched exactly the detection of cancer by standard diagnostic methods. All men with diagnosed prostate cancer before CTC testing had CTC, and screened men with PSA-marker positive CTC had a positive prostate-specific diagnostic follow-up test.

The high accuracy of the ISET-CTC test combined with the 97% sensitivity and 99% specificity of the PSA-marker presence on prostate cancer cells, suggests an estimated positive predictive value (PPV) of 99% and a negative predictive value (NPV) of 97% for this novel screening test.

### Prostein (P501S)

ICC staining with Prostein in a subgroup of patients (*n* = 8) was equivalent to the staining by PSA markers in all except one case (Tables 1, 2).

### Inflammatory Prostate Conditions and Negative Controls

No CTC but atypical inflammatory cells were found by the cytology-based ISET®-CTC technology in two out of the 27 men screened for CTC (group ED). These men presented with prostate symptoms and slightly elevated PSA-levels (Table 2, group ED4). The ISET®-CTC-test accurately demonstrated a non-cancerous inflammatory condition in these patients. As a negative control, we undertook PSA-marker-testing on two females with breast or ovarian cancer, and the result was negative as expected (Table 2, negative control).

### Long-Term Follow-Up

For a subset of patients in this study, long-term data was available which included regular CTC-testing results, treatment details, and disease progression.

ISET®-CTC-test results before and after prostate cancer diagnosis were available for 6 men in the early detection group (Table 2), with PSA-marker-positive-CTC apparent 6 months-to-3.5 years before prostate cancer diagnosis. Five of these patients received advice and treatments of integrative therapies, and one underwent prostatectomy. CTC-count reduced over-time in those following treatment, evident by repeated ISET®-CTC-tests, suggesting a decreased malignant potential. One of the six patients however, followed the watch-and-wait approach, not receiving advice on preventative therapies, and unfortunately developed metastatic-bone-lesions 3.5 years after the initial CTC-test, with PSA(+) CTC (2.2 CTC/ml) (Table 2).

In this small number of patients where long-term monitoring by repeated CTC-testing was available, CTC-count by ISET® was consistent with cancer progression and treatment effectiveness (Table 2).

## Discussion

Our study suggests the combination of ISET®-CTC and ICC-prostate-marker testing to be a highly sensitive and accurate screening test for prostate cancer, with 97% sensitivity and 99% specificity. All patients with confirmed prostate cancer tested positive for prostate-specific CTC, and all screened males with prostate-specific CTC had an affirmative PSMA-PET scan result, highly suggestive of prostate cancer. Half of the screened men with positive PSMA-PET scan results underwent biopsies, confirming the diagnosis of prostate cancer in each case.

Our findings are in line with a recently published study using a similar filtration-based isolation of CTC method, the Parsortix system (ANGLE Guildford, UK; which combines CTC and ICC) to screen men for the likelihood of prostate cancer (46). The study concluded that the combination of CTC and a 12-gene panel screening test was able to predict the presence of prostate cancer in subsequent biopsies with over 90% accuracy (46).

In contrast, the PSA-blood-test failed to match the abnormal CTC test results in 83% of study participants, including 73% of men with prostate cancer (group PC), and 88% of the men screened (group ED) with positive PSA-marker-CTC and early prostate cancer diagnostic results.

Importantly, the detection of ICC-PSA-negative CTC in screened men allowed the early detection of other types of cancer, namely renal cancer, or B-cell lymphoma. Additionally, the new combination of CTC and ICC testing provided evidence of the presence of relapses or secondary cancers such as bladder, bowel, thyroid, tongue cancer, and metastasis in bone and lymph nodes in men with prostate cancer.

The findings are in line with earlier studies, whereby the CTC count was directly related to the risk of cancer detection and cancer status (9, 10, 12).

The early detection of cancer is paramount to enable medical interventions that will reduce the risk of cancer morbidity and mortality. Early detection allows more time and treatment options in addition to active surveillance, before surgery and/or radiation therapy.

There is evidence that preventative integrative and nutritional therapies can reduce the CTC count and therefore cancer risk, outlined elsewhere in more detail (9). Hyperthermia treatment has also shown to improve treatment effectiveness of other therapies e.g., chemotherapy, by an average of 40–80% (47, 48), and may be considered for patients with higher CTC-counts.

With its estimated sensitivity of 97% and specificity of 99%, this study's combination of ISET®-CTC and ICC-PSA-marker testing offers a unique opportunity to replace the unsatisfactory PSA-blood-test as a screening test.

Due to the PSA-blood-test's high false negative rate of 85.5%, and the low 25% true positive rate (3), the PSA-blood-test is of limited use for prostate cancer screening. On the other hand the low sensitivity of the PSA-blood-test results in many men have led to unnecessary procedures that carry with them high complication rates, exposure to toxic treatments and debilitating post-surgical complications (2).

In current clinical practice, the limitations of the PSA-blood-test are being addressed by performing multi-parametric magnetic resonance imaging (MRI) before biopsies to decrease the detection of non-significant prostate cancer and to improve the detection of significant prostate cancer (49, 50). However, a more accurate screening test than the PSA-blood-test may improve the prediction of prostate cancer by itself, and would reduce the necessity of MRI imaging before biopsy.

A strength of our study was the use of one of the most sensitive and accurate CTC-screening tests currently available, the cytology-based ISET®-CTC-test (14), which clearly distinguishes cancer cells from atypical inflammatory cells, making it possible to accurately predict a risk of cancer or an inflammatory condition, such as prostatitis (16). Almost every participant with an inflammatory condition in our larger study involving 2,000 patients had no CTC upon screening (9), while marker-based CTC-tests such as CellSearch® cannot distinguish between CTC and atypical inflammatory cells, as both types of cells can have the cell-surface markers, leading to many false positives, and false negatives (20, 21, 51).

Confirmatory prostate cancer diagnosis by gold-standard biopsy was applicable for 40 out of 47 participants and was performed in 73% of those. These included all men with previously diagnosed prostate cancer (*n* = 20), and a proportion of men screened with positive PSA-marker stained CTC (*n* = 9/20 men, 45%).

Our study has some limitations. First, not all screened men with positive PSMA-PET scans agreed to biopsies for confirmatory diagnosis of prostate cancer at the time of the study. However, the PSMA-PET scan has been shown to be a highly accurate, less invasive diagnostic tool with high sensitivity and specificity, useful in the detection of prostate cancer, especially in lower risk men reluctant to undergo biopsy (35–37, 39–41).

Second, prostate-specific diagnostic testing was not available for a small number of screened men (*n* = 5/25) included in this study. However, these men underwent other relevant follow-up testing as advised by their doctor, and in line with their non-prostate specific CTC test results, which enabled the detection of other cancers in two of the five men.

Third, we tested only a small group of patients with the ICC-Prostein-marker, therefore findings are preliminary and warrant further studies.

Despite the limitations, our results indicate that combining the ISET®-CTC-test with other ICC-tumor-cell markers for the early detection of other cancers is promising and warranted. Early detection will be particularly important for aggressive silent cancers, such as ovarian or pancreatic cancer, as time is of the essence for long-term quality of life and survival. Only 15% of ovarian cancers are currently detected in stage 1 with the available screening-tests, and preventive oophorectomy results in premature menopause and aging (52, 53).

Pancreatic cancer is specifically deadly (16% 5-year-survival-rate) when detected late. In contrast, if detected early, treatment by surgery can improve 5-year survival rates substantially to 61% (54). However, only a small percentage of pancreatic cancer is detected early, highlighting the need for better screening tests.

## Conclusions

This study provides evidence for the combined ISET®-CTC and ICC-PSA-marker testing to be a more accurate screening test than the PSA-blood-test alone. This novel combination of tests has an estimated positive predictive value of 99% and a negative predictive value of 97%, warranting further studies to evaluate the new test's potential for prostate cancer screening on a population level. The test allows early detection of prostate cancer, as well as other types of cancer, providing the opportunity for early intervention. The new screening test combination also allowed long-term monitoring of patients diagnosed with prostate cancer, providing insight to relapse or metastasing potential.

The ISET®-CTC-test alone allows monitoring of treatment effectiveness in patients with cancer of any type and offers an opportunity for the early detection of cancer risk. The combination of the ISET®-CTC and organ-specific cell markers provides an exciting future for cancer screening.

## Data Availability Statement

All datasets generated for this study are included in the article.

## Ethics Statement

The studies involving human participants were reviewed and approved by NIIM HREC, National Institute of Integrative Medicine, Melbourne, Australia. The patients/participants provided their written informed consent to participate in this study.

## Author Contributions

KR established and oversaw ISET®-CTC-testing and ICC prostate marker testing at the NIIM lab, with assistance from cytologist SM and post-doctoral researcher TT. Medical practitioners PE and AS provided patients, and patient data for the study. KR collated and analyzed the data and wrote the manuscript with contributions from co-authors. All authors read and approved the final version.

## Conflict of Interest

The authors declare that the research was conducted in the absence of any commercial or financial relationships that could be construed as a potential conflict of interest.
